# Finite element modelling predicts changes in joint shape and cell behaviour due to loss of muscle strain in jaw development

**DOI:** 10.1016/j.jbiomech.2015.07.017

**Published:** 2015-09-18

**Authors:** Lucy H. Brunt, Joanna L. Norton, Jen A. Bright, Emily J. Rayfield, Chrissy L. Hammond

**Affiliations:** a Schools of Physiology and Pharmacology and of Biochemistry, University of Bristol, BS8 1TD Bristol, United Kingdom; b School of Earth Sciences, University of Bristol, BS8 1RJ Bristol, United Kingdom

**Keywords:** Zebrafish, Biomechanics, Strain, Cells, Joint morphogenesis, Finite element

## Abstract

Abnormal joint morphogenesis is linked to clinical conditions such as Developmental Dysplasia of the Hip (DDH) and to osteoarthritis (OA). Muscle activity is known to be important during the developmental process of joint morphogenesis. However, less is known about how this mechanical stimulus affects the behaviour of joint cells to generate altered morphology. Using zebrafish, in which we can image all joint musculoskeletal tissues at high resolution, we show that removal of muscle activity through anaesthetisation or genetic manipulation causes a change to the shape of the joint between the Meckel's cartilage and Palatoquadrate (the jaw joint), such that the joint develops asymmetrically leading to an overlap of the cartilage elements on the medial side which inhibits normal joint function. We identify the time during which muscle activity is critical to produce a normal joint. Using Finite Element Analysis (FEA), to model the strains exerted by muscle on the skeletal elements, we identify that minimum principal strains are located at the medial region of the joint and interzone during mouth opening. Then, by studying the cells immediately proximal to the joint, we demonstrate that biomechanical strain regulates cell orientation within the developing joint, such that when muscle-induced strain is removed, cells on the medial side of the joint notably change their orientation. Together, these data show that biomechanical forces are required to establish symmetry in the joint during development.

## 1 Introduction

The development of reciprocal interlocking joints at cartilage elements is central to ensuring normal skeletal function (Nowlan et al., 2010). Processes that disrupt joint shape formation can cause abnormal loading and joint function (Michaeli et al., 1997), e.g. hip shape correlates strongly with risk of osteoarthritis (Jacobsen and Sonne-Holm, 2005). The initial formation of the cartilage template from mesenchymal cell condensations, mostly replaced by bone, is well understood (DeLise et al., 2000; Thorogood and Hinchliffe, 1975). However, how the early joint structures undergo morphogenesis to form their mature shape remains less clear (Pacifici et al., 2005).

**Fig. 1:**
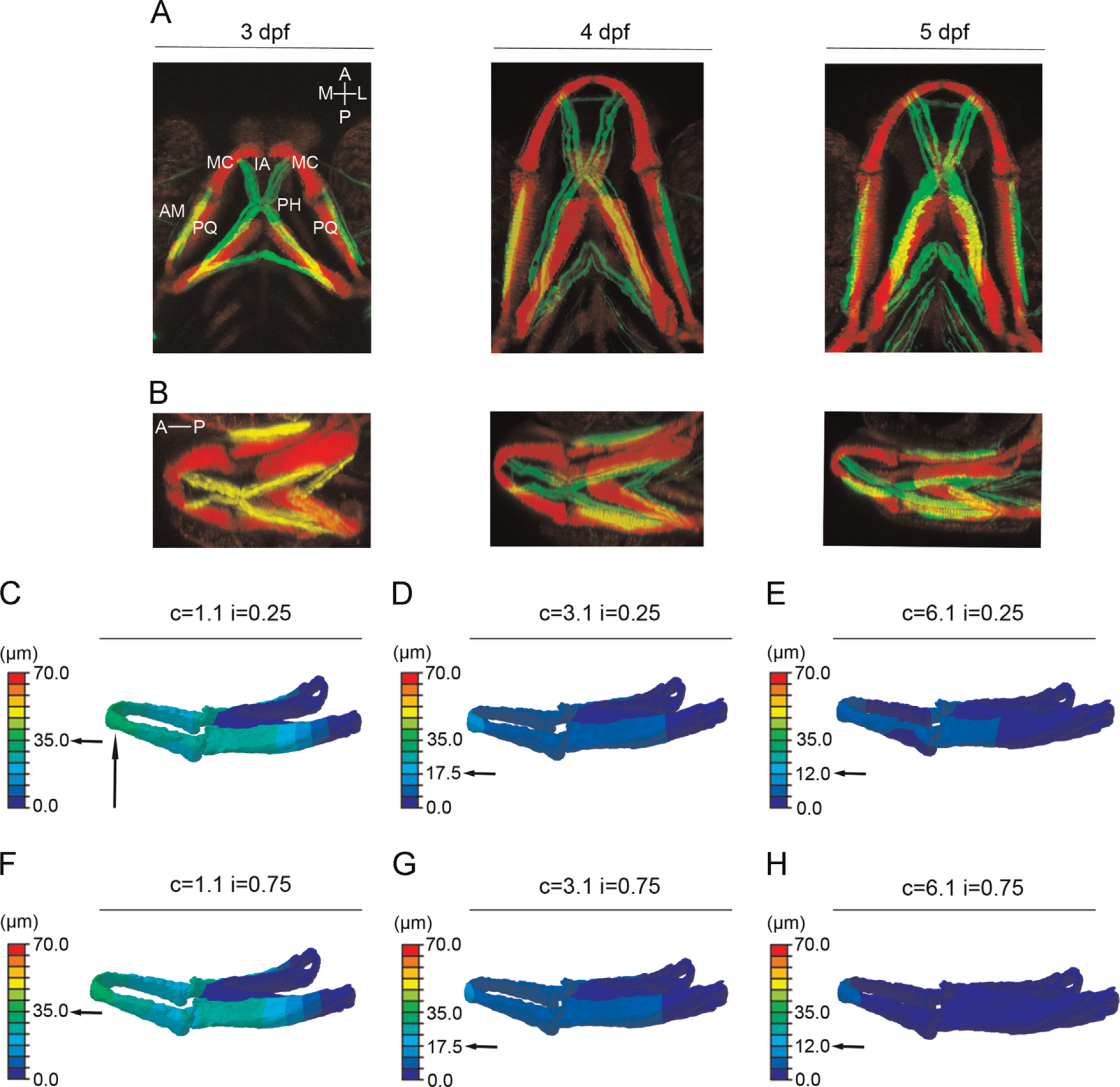
FE-model simulating jaw displacement in a 5 day old fish (5 dpf) for different cartilage and interzone Young's moduli. (A, B): Confocal image stacks of the ventral (A) and lateral (B) zebrafish jaw at 3, 4, and 5 dpf with cartilage labelled by *Tg(Col2a1aBAC:mcherry)* and muscle labelled by *Smyhc:GFP*. AM=adductor mandibulae, IA=intermandibularis anterior, PH=protractor hyoideus, MC=Meckel's cartilage, PQ=palatoquadrate, M=medial, L=lateral, A=anterior, P=posterior. (C–H): Jaw displacement (open to closed in µm) is marked on the jaw; recorded using the colour key. Each model (C–H) has a different combination of cartilage (*c*=1.1, 3.1, or 6.1 MPa) or interzone (*i*=0.25 or 0.75 MPa) properties. Horizontal black arrow highlights the value of jaw displacement at the tip of the Meckel's cartilage (represented by the vertical black arrow).

**Fig. 2:**
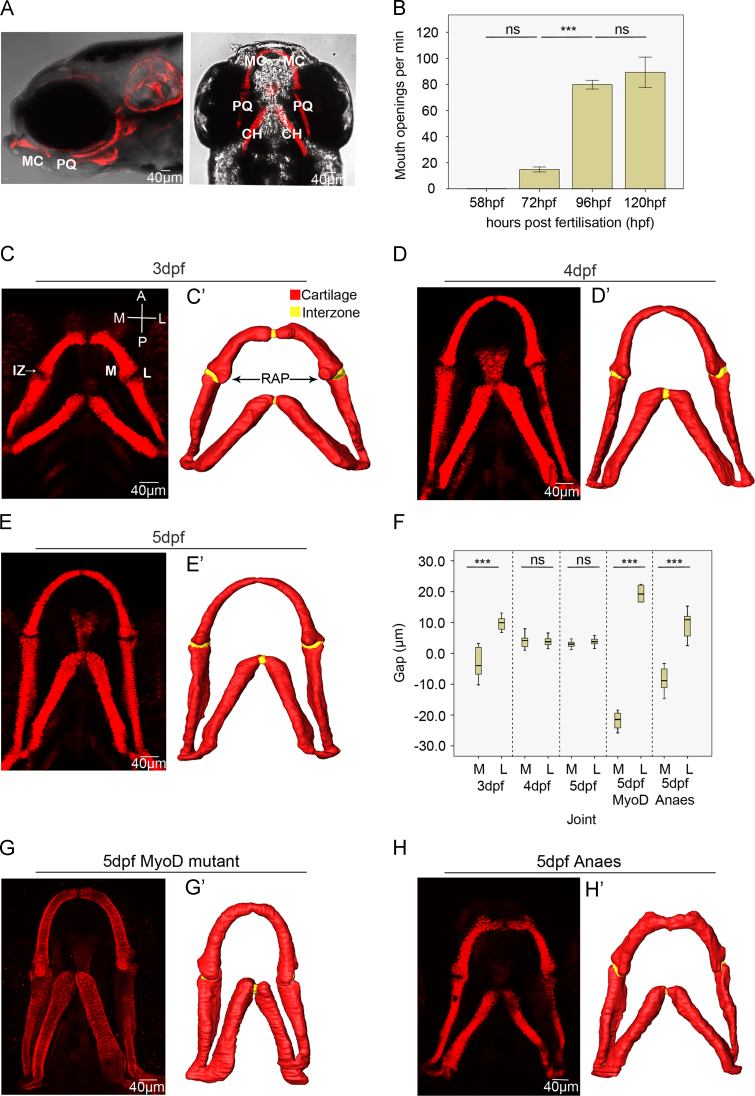
Changes to jaw joint morphology between 3 and 5 dpf and the effect of muscle activity on joint shape. (A): Brightfield lateral and ventral view of 5 dpf zebrafish expressing *Tg(Col2a1aBAC:mcherry)* cartilage marker MC=Meckel's cartilage, PQ=palatoquadrate, CH=ceratohyal. (B): The number of mouth openings per minute at 58 h post-fertilisation (hpf), (*n*=10), 72 hpf (*n*=6), 96 hpf (*n*=6) and 120 hpf (*n*=6). ns= not significant, **^⁎⁎⁎^**=*P*≤0.001. (C, D, E): Confocal image stacks of ventral zebrafish jaws at 3 (C), 4 (D), and 5 dpf (E), marked with a *Tg(Col2a1aBAC:mcherry)* cartilage marker. The medial and lateral sides of the elements are labelled M and L and the anterior and posterior surfaces of the elements are labelled A and P. IZ marks the interzone. (C’, D’, E’): 3D *Avizo* reconstructions from the confocal datasets: cartilage marked red, interzone marked yellow. RAP denotes the retroarticular process of the Meckel's cartilage (F): Box and whisker plot showing the interval between the anterior MC and posterior PQ elements on the medial and lateral side of the joints at 3 dpf (*n*=12), 4 dpf (*n*=16), 5 dpf (*n*=13) and in 5 dpf *myod* (*n*=4) and 5 dpf anaesthetised zebrafish (*n*=8), ns=not significant, ^⁎⁎⁎^=*P*≤0.001. Negative measurements indicate an overlap of the anterior MC and posterior PQ elements. (G, H): confocal image stacks of the ventral jaws of 5 dpf *myod* mutant (G) and 5 dpf anaesthetised zebrafish (H) marked by *Tg(Col2a1aBAC:mcherry)* cartilage marker. (G’, H’) 3D *Avizo* reconstructions from the confocal datasets. (For interpretation of the references to color in this figure legend, the reader is referred to the web version of this article.)

Zebrafish, with relevant fluorescent transgenic lines (Apschner et al., 2014; Hammond and Moro, 2012), allow dynamic imaging of the musculoskeletal system at cellular resolution. Zebrafish are, therefore, a useful model to examine how mechanical loading from muscle impacts on cartilage behaviour. By 48 h post-fertilisation (hpf), mesenchymal cells have condensed to form the mandibular arches (Eames et al., 2013; Kimmel et al., 1995). At 53–55 hpf, the cartilaginous elements of the Meckel's cartilage (MC), palatoquadrate (PQ) and ceratohyal appear, as does the adductor mandibulae jaw musculature, with the intermandibularis anterior and protractor hyoideus, identifiable by 62 hpf (Schilling and Kimmel, 1997). Larval zebrafish use the protractor hyoideus to constrict the buccal chamber of the jaw and adductor mandibulae to close the mouth (Diogo et al., 2008; Hernandez et al., 2002). The joint between the MC and PQ (Figs. 1A and 2A) is described as the jaw joint in (Talbot et al., 2010) and referred to as such hereafter. In the joint structure, the retroarticular process (RAP) of the MC protrudes ventrally to interlock with the PQ (Miller et al., 2003), typically cavitation of this joint occurs at around 7 dpf (http://zfatlas.psu.edu/).

Many studies have linked absence of muscle activity with abnormal joint shaping and fusions of articular surfaces in long bones. Early studies used Decamethonium Bromide and botulinum toxin to generate paralysis in chick models; leading to a flattening of articular surfaces and a failure of joint cavitation (Drachman and Sokoloff, 1966; Murray and Drachman, 1969). More recently, (Roddy et al., 2011b) found that rigid paralysis of chicks during early development caused the knee joint to appear flattened. Additionally, genetic models in mice, such as Splotch mutants that lack limb muscle, exhibit abnormal limb joint shaping (Kahn et al., 2009; Nowlan et al., 2010). While muscle force has been shown to be necessary for normal joint morphogenesis and chondrocyte intercalation (Shwartz et al., 2012), it remains largely unclear how cells within the joint interpret such forces to bring about changes in behaviour. Little is known concerning the effects of paralysis on jaw joint morphology, though the temporomandibular joint region shows signs of adaptation when the mechanical environment is altered (Enomoto et al., 2014; McNamara and Carlson, 1979).

Finite element (FE) models simulating the biophysical response to muscle-induced loading have been used to investigate joint development, endochondral ossification, and joint development, including developmental dysplasia of the hip (DDH) (Carter and Wong, 1988; Heegaard et al., 1999; Nowlan et al., 2008; Roddy et al., 2011a; Shefelbine and Carter, 2004). Thus far, developmental FE-models have focused primarily on the femur, particularly in humans and chick. Whilst there are a handful of studies using FE to deduce the mechanical performance of extant shark jaws (Ferrara et al., 2011) and other jawed vertebrates (Rayfield, 2007), FE-modelling has not been used to explore the mechanobiology of the developing zebrafish jaw pre-cavitation.

Here, we document the process of joint morphogenesis in wild type zebrafish jaws from the time of first muscle activity to generation of the refined interlocking joint shape. Then, by removing muscle activity pharmacologically and genetically, we quantify the timing and extent of the response to lack of muscle activity on joint morphogenesis. Using FE analysis (FEA) we identify the locations of muscle-induced strain acting on the zebrafish jaw cartilage throughout normal joint morphogenesis. To understand the mechanobiological changes that underpin joint shape we quantify differences in cellular orientation between wild type, anaesthetised and mutant models.

## 2 Methods

### 2.1 Zebrafish husbandry/zebrafish lines

Zebrafish were maintained as described (Westerfield, 2000). All zebrafish experiments were approved by the local ethics committee and the Home Office (Project license number 30/2863). The *Tg(Col2a1aBAC:mcherry)* zebrafish line has been previously described (Hammond and Moro, 2012; Hammond and Schulte-Merker, 2009) and labels all chondrocytes (Fig. 2A). The line *Tg(smhyc:EGFP)i104* labels all slow twitch muscle fibres (Elworthy et al., 2008). *myod*^fh261^ mutants have been previously described and lack craniofacial muscle, with the exception of the sternohyoideus (Hinits et al., 2011).

### 2.2 Counting mouth movements and measuring jaw displacement

Zebrafish were anaesthetised with MS222 (Tricaine methanesulfonate), (Sigma) and mounted laterally onto coverslips in 3% agarose. The agarose surrounding the head was removed and Danieau buffer (Westerfield, 2000) flushed over the coverslip until jaw movements resumed. The number of mouth movements in 1 min was recorded from at least six fish per timepoint. Movies of jaw movements were made using fish labelled with *Tg(Col2a1aBAC:mcherry)* on a Leica SP8 confocal microscope. Jaw movement was quantified as the positional change at the anterior end of the MC measured (µm) from individual movie frames.

### 2.3 Anaesthetisation

**Fig. 3:**
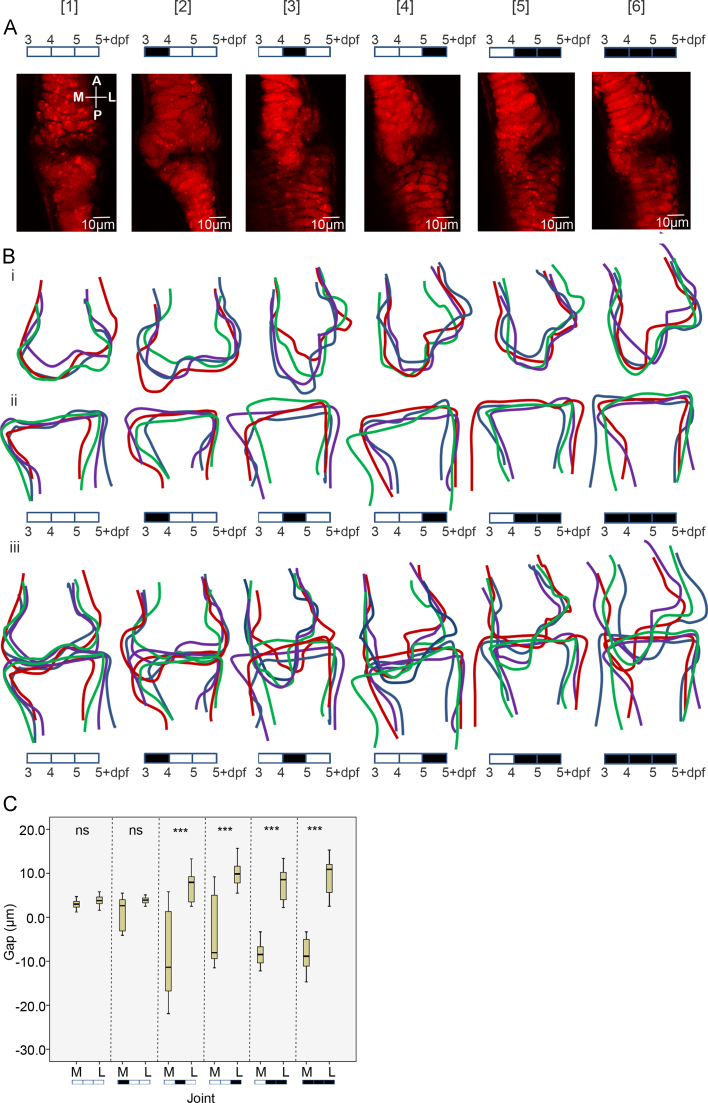
Imaging and quantification of joint shape. (A); zebrafish 5+ dpf jaw joints labelled with the *Tg(Col2a1aBAC:mcherry)* cartilage marker, anaesthetised with MS222 for varying time periods between 3 and 4 [2], 4 and 5 [3], 5− and 5+ [4], 4–5+ [5] and 3–5+dpf [6]. (5+dpf=128 hpf). A black box on the 3–5+dpf timeline indicates anaesthetisation by MS222 and an empty box indicates no MS222 treatment. The medial and lateral side of the elements are labelled M and L and the anterior and posterior surfaces of the elements are labelled A and P. (B): Outline of the 5+ dpf jaw joint shape after each anaesthetic treatment, anterior Meckel's cartilage joint element (Bi), posterior Palatoquadrate joint element (Bii), and the extent of the joint element overlap (Biii) (*n*=4, each colour representing an individual fish). All outlines are to the same scale. (C): Box and whisker plot of the interval between the anterior MC and posterior PQ elements on the medial and lateral side of the joints at 5+dpf (*n*=13, 13, 10, 10, 16, 16, 18, 16, 8, 8, 12, 14, 8, 8), ns=not significant, ^⁎⁎⁎^=*P*≤0.001. Negative measurements indicate an overlap of the anterior MC and posterior PQ elements.

Wild type zebrafish were anaesthetised in MS222 in Danieau buffer. The solution was refreshed twice daily. Treated fish were observed to ensure that anaesthesia was effective and jaw movement had ceased, but heart rate was normal. Jaw element and joint morphology were imaged and quantified at 3, 4 and 5 days post-fertilisation (dpf). A second set of ‘wash-out’ experiments were performed where the anaesthetic was replaced with fresh Danieau buffer at different time points between 3 dpf and 5 dpf (72–128 hpf; 128 hpf referred to as 5+dpf) to allow fish to recover muscle activity. Fish were washed in successive changes of Danieau buffer, until fish recommenced movement. Six different trials were performed, (Fig. 3A): [1] a control without anaesthetic treatment; [2] anaesthesia from 3–4 dpf (72–96 hpf), [3] anaesthesia from 4–5 dpf (96–120 hpf), [4] anaesthesia from 5–5+dpf (120–128 hpf), [5] anaesthesia from 4–5+dpf (96–128 hpf); preceded or followed by normal conditions, or [6] constant anaesthesia from 3–5+dpf (72–128 hpf). The resulting joint shape was imaged at 5+dpf (128 hpf) in all trials.

### 2.4 Jaw imaging

Confocal images of approximately 100 slices at 1.3 µm intervals were taken of jaws at 3, 4 and 5 dpf labelled with *Tg(Col2a1aBAC:mcherry)* using an SP8 Leica confocal.

### 2.5 Immunohistochemistry

Immunohistochemistry was performed as previously described (Hammond and Schulte-Merker, 2009) II-II6B3- (type II collagen, Developmental Studies Hybridoma Bank) was added at 1:200 and A4.1025 (skeletal myosin (Dangoor et al., 1990)) was added at 1:5. Larvae were viewed by confocal and images were processed using Leica LAS AF Lite software and ImageJ (Schneider et al., 2012).

### 2.6 Outline traces

The curve drawing tool in powerpoint was used to produce the outlines of the jaw joint labelled with *Tg(Col2a1aBAC:mcherry)* from stacks of confocal images; each specimen was drawn in a separate colour.

### 2.7 Interzone measurements

The interval between the MC and PQ cartilage elements of the jaw joint on their medial and lateral sides (typically the smallest and largest gaps between cartilage across the joint, respectively) were measured (from both the left and right joints) from confocal images of the jaw using Leica LAS AF Lite software. Negative values were recorded for overlapped cartilage elements.

### 2.8 Geometry and Finite Element Analysis

A 3D 512×512 pixel resolution representation of the stained larval zebrafish jaw at 3, 4 and 5 dpf was produced using confocal microscopy. These datasets were imported into *Avizo* (Version 7.0.0 FEI Visualization Science Group) for material segmentation and digital reconstruction to produce 3D models of the cartilage elements. The models were imported into *Hypermesh* (Version 10, Altair Engineering) to generate a solid mesh of approximately 1.5 million tetrahedral linear elements (Supp. Fig. 1, Supp. Table 1). Due to the absence of known zebrafish cartilage material properties, a range of Young's moduli for the cartilage and interzone material were tested on the 5 dpf model to determine which properties generated jaw displacements, (measured from the tip of the MC, Fig. 1C), that best matched those observed in Table 1. When 50% of maximum calculated muscle force was applied to the models, all models that had a cartilage Young's modulus of 1.1 MPa fell within a physiological range of jaw displacement (Table 1). Young's modulus of 1.1 MPa for cartilage (as previously reported for unmineralised embryonic murine cartilage (Tanck et al., 2004)) and 0.75 MPa for the interzone were chosen for the models (Fig.1). These material properties were assigned hereafter, with a Poisson's ratio of 0.25, previously described for embryonic unmineralised murine cartilage (Tanck et al., 2000).

### 2.9 Muscle force measurement and calculation

To model strains acting on the jaw, we calculated muscle forces during jaw opening (protractor hyoideus and intermandibularis) and closing movements (adductor mandibulae). The *Tg(smhyc:EGFP)i104* (slow twitch skeletal muscle) transgenic line was used to determine muscle attachment points. However, as jaw muscles are composed of a mixture of fast and slow twitch fibres (Hernandez et al., 2005), we performed immunohistochemistry for the antibody A4.1025, which detects both slow and fast twitch fibres (Dangoor et al., 1990), allowing us to count the actual number of muscle fibres in each muscle (Table 2).

Cross-sectional area of each fibre was used to determine the force that each muscle group exerts at 40 nN/µm^2^ (Table 2), the force for skeletal larval zebrafish muscle previously described (Iorga et al., 2011). Muscle attachments were added to the FE-models for mouth closure (adductor mandibulae) and opening (protractor hyoideus and intermandibularis) using confocal datasets for reference for attachment sites (Supp. Figs. 1, 1A and B). Models were constrained from movement at the junction between the hyosymplectic and palatoquadrate where the jaw attaches the skull and the ceratohyal (Supp. Table 1, Supp. Fig. 1). The left and right side of the models were constrained in *y* and *z*, whilst the ceratohyal was constrained in *x*, *y* and *z*. The geometrically linear models were imported from *Hypermesh* into *Abaqus* FE-software (v6.10.2 Simulia, Dassault Systèmes) for analysis. Maximum principal strain, (tension), and minimum principal strain (compression) were recorded.

### 2.10 Cell orientation measurements

Cell orientation analysis on the Meckel's cartilage joint was performed using confocal images of joints labelled with II-II6B3 antibody (expanded methodology in Supp. Fig. 2). Cells from three joints per condition were identified by thresholding in ImageJ (Supp. Table 2, Supp. Fig. 2B). The angle of the joint from the horizontal axis was recorded (Supp. Fig. 2B). An automated readout of cell orientation was produced (Supp. Fig. 2C). These data were exported into Excel, and angles corrected in relation to the joint axis, (Supp. Fig. 2C). Joint cell orientation was plotted in circular histograms using PAST software (Hammer et al., 2001).

### 2.11 Statistics

Statistics were performed using SPSS software. Kruskal–Wallis tests were performed to compare the size of the cartilage interval at the medial and lateral edges of the joint following each anaesthesia treatment (Figs. 2F and 3C). The Kruskal–Wallis test was used to make multi-comparisons between non-normal data.

## 3 Results

### 3.1 Jaw mobility

The first stage at which jaw opening was reliably present was 72 hpf (Fig. 2B). Subsequently, there was a significant increase in jaw motility between 72 and 96 hpf, whereas there was no significant difference between 96 hpf and 120 hpf. Mouth opening had therefore reached its maximal frequency by 96 hpf.

### 3.2 Jaw morphology

Confocal imaging and the resulting 3D digital reconstructions show that the interval between cartilage elements (the joint interzone) changes in morphology across the time period in the control fish (Fig. 2). At 3 dpf there is a smaller interval between the Meckel's cartilage (MC) and the palatoquadrate (PQ) on the medial side than on the opposing lateral side (Fig. 2C, C’), i.e. the joint interzone is asymmetrically shaped at 3 dpf. By 5dpf the interzone is similar in width across its extent (Fig. 2 C–E’). To quantify this change in morphology we measured the interval between the MC and the PQ at the medial and lateral side at each time point (Fig. 2F). Kruskal–Wallis tests showed a significant difference in the relative size of the interzone at its greatest and smallest extent at 3 dpf, which decreases over time, such that by 5 dpf the difference in the size of the interval across the joint is no longer significant and the interval is roughly uniform across its extent. *myod* mutants, (which lacks all jaw movement), have a similar morphology to wild type at 3 dpf (Supp. Fig. 4) but a significant difference between the cartilage element interval on the medial and lateral side at 5 dpf compared to 5 dpf wild type (Fig. 2F, G and G’). We also tested the size of the cartilage interval of the *myod* compared to younger wild type fish; *myod* fish at 5 dpf retain a joint interzone shape comparable with that of a 3 day old wild type (Fig. 2F and G, Supp. Table 3).

Fish anaesthetised from 3–5 dpf have a significant difference in the extent of the cartilage interval between the medial and lateral side compared to wild type fish at 5 dpf (Fig. 2F, H, and H’), but are not significantly different to the *myod* mutants (Fig. 2F–H, Supp. Table 3). Therefore, it is the activity rather than the presence of muscle which leads to joint morphogenesis.

### 3.3 Variable anaesthesia and joint morphology

Kruskal–Wallis tests show that anaesthesia from 3 to 4 dpf does not lead to any significant difference in joint spacing between the medial and lateral sides at 5 dpf relative to control fish (Fig. 3A1–2, C). Anaesthesia from 4 to 5 dpf, 4 to 5+ dpf, 3 to 5+ dpf or 5 dpf alone significantly alters the size of the interzone between the medial and lateral sides (Fig. 3A1, 3–6, C).

Outline traces indicate that the joint surface of the MC is more plastic than the surface of the palatoquadrate (Fig. 3Bi, ii). Anaesthesia at 3–4 dpf alone did not lead to a dramatic shape change of the MC (Fig. 3Bi–iii), as reflected in the interzone interval analysis (Fig. 3C), however, later anaesthesia leads to a change in joint morphology such that the MC approaches or overlaps the surface of the PQ on the medial side (Fig. 3Biii).

### 3.4 Functional strains within the developing cartilage jaw

**Fig. 4:**
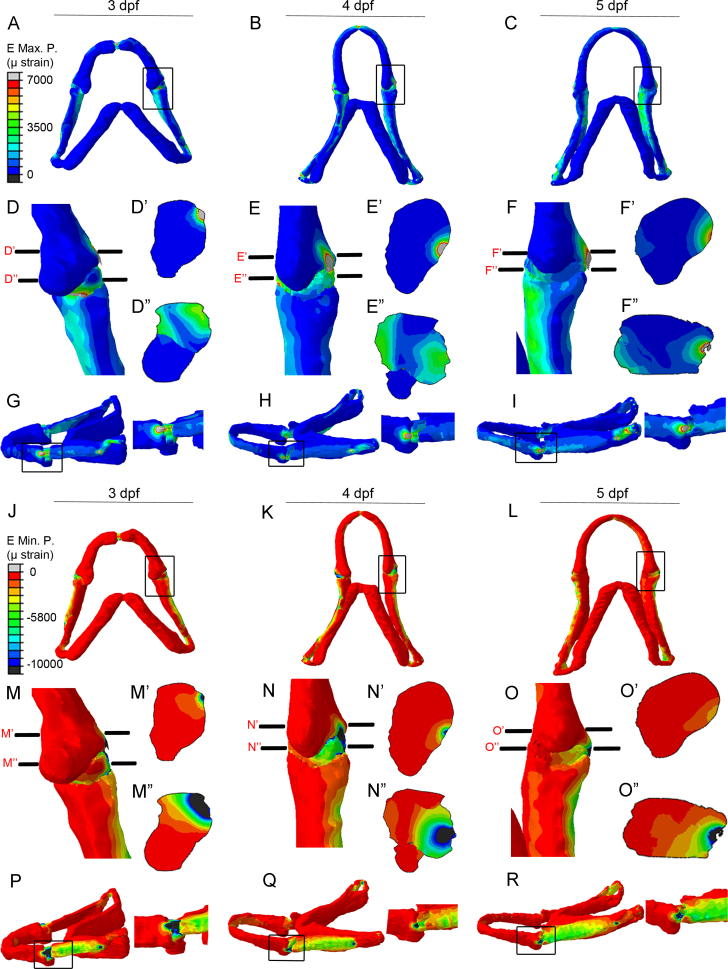
Finite Element Analysis (FEA) showing location of strains at the wild type jaw joint. (A–I): Finite element models of maximum principal strain (*E*_max_, tension) created from confocal stacks for 3, 4, and 5 dpf zebrafish jaws. Ventral views of the jaw (A–C), with an enlarged image of the joint from the boxed area (D–F). Proximal–distal view of the joint (D’–F’) and proximal–distal view of the interzone (D’’–F’’), (as illustrated as slices through the joint in D–F). Lateral views of the jaw (G–I) with an enlarged image of the joint from the boxed area. (J–R): Finite Element models of minimum principal strain (*E*_min_, compression) created from confocal stacks for 3–5 dpf zebrafish jaws. Ventral views of the jaw (J–L), with an enlarged image of the joint from the boxed area (M–O). Proximal–distal view of the joint (M’–O’) and proximal–distal view of the interzone (M’’–O’’), (as illustrated as slices through the joint in M–O). Lateral views of the jaw (P–R) with an enlarged image of the joint from the boxed area.

**Fig. 5:**
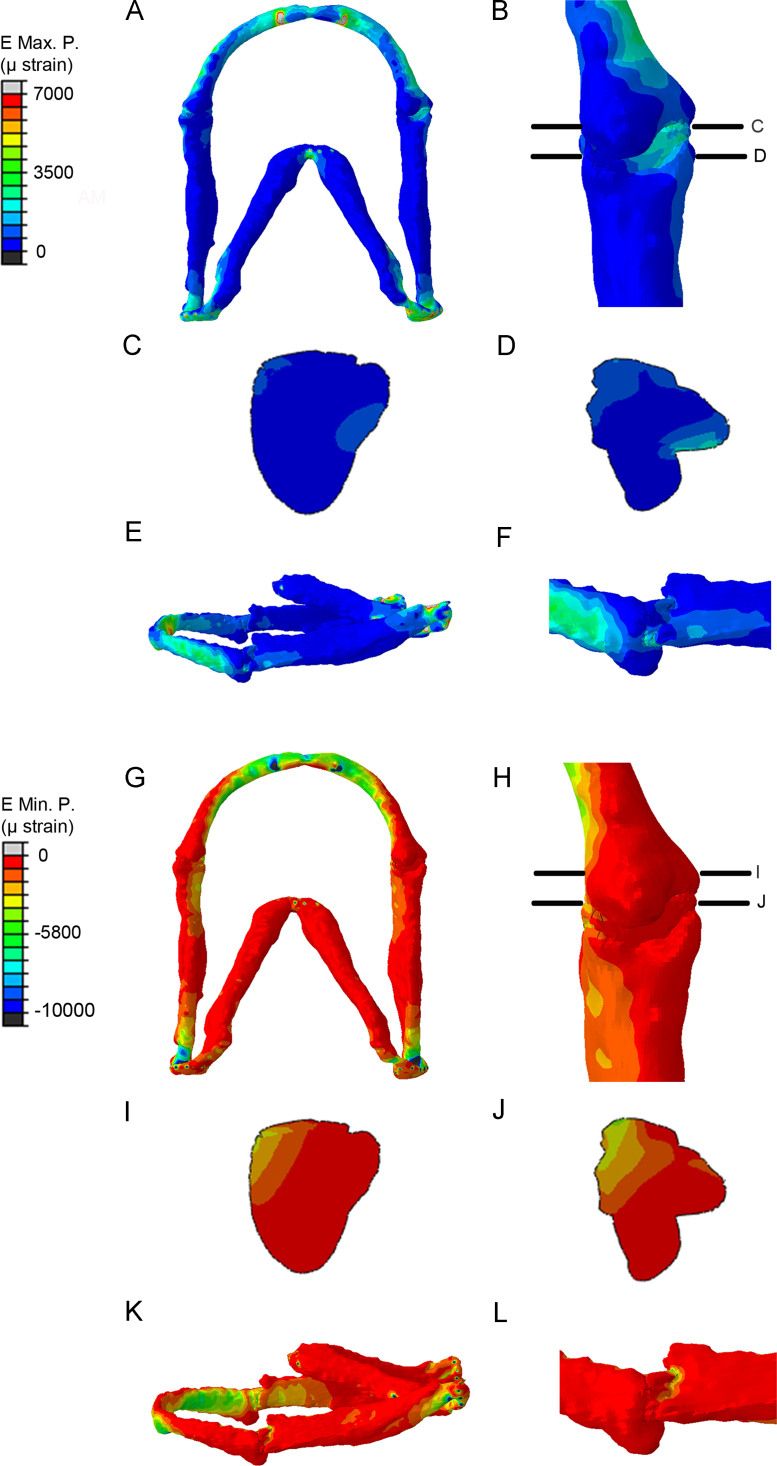
FE-model simulation of maximum and minimum principal strains in the 5 dpf wild type zebrafish jaw during jaw opening. (A–F): Maximum principal strain in (A) ventral jaw view; (B) ventral joint view; (C) proximal–distal view through the joint (illustrated on (B)); (D) proximal–distal view through the interzone (illustrated on (B)); (E) lateral jaw view; (F) lateral joint view. (G–L): minimum principal strain in (G) ventral jaw view; (H) ventral joint view; (I) proxima–distal view through the joint (illustrated on (H)); (J) proximal–distal view through the interzone (illustrated on (H)); (K) lateral jaw view; (L) lateral joint view.

FE-models demonstrate maximum principal strains present upon jaw closure (generated by adductor mandibulae muscle contraction) (Fig. 4A–I) and minimum principal strains at 3–5 dpf (Fig. 4J–R). We also generated models of jaw opening via contraction of the protractor hyoideus and the intermandibularis (Diogo et al., 2008) (Fig. 5). From these models it is apparent jaw closure causes maximum principal strains to be located medially and laterally at the interzone and minimum principal strains are located laterally (Fig. 4 D’’–F’’, M’’–O’’). During jaw opening, compressive strains (minimum principal strains) are recorded on the medial side of the MC joint and at the interzone (Fig. 5G–J). Relevant interzone strains were always between +3500 µstrain (maximum) and −5000 µstrain (minimum), in line with other models (Nowlan et al., 2008), (Supp. Fig. 5).

### 3.5 Joint cell orientation

**Fig. 6:**
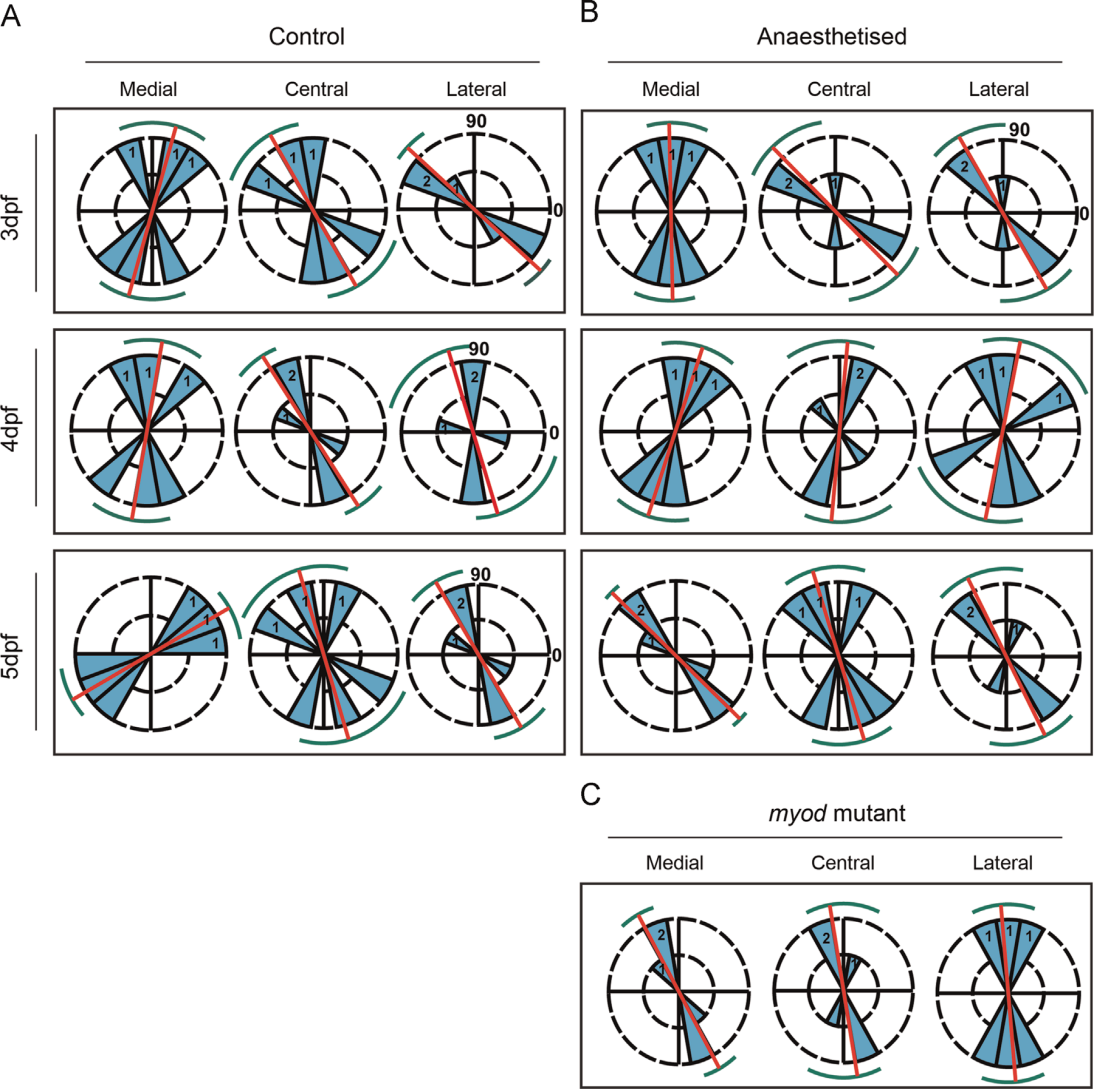
Cell orientations in wild type, anaesthetised and *myod* mutant zebrafish jaw joints. Orientation angle of chondrocytes in (A) 3–5 dpf control; (B) 3–5+ dpf anaesthetised; and (C) *myod* mutant zebrafish in the Meckel's cartilage element of the joint, plotted on circular histograms (rose plots), where 0° lies on the medial side of the joint and 180° at the lateral side of the joint. *n*=3 joints per experimental condition (1 or 2 refers to number of joints per blue wedge). The number of cells analysed per condition are listed in Supp. Table 2. Histogram bins equal 20°. The red line marks mean orientation and the green line marks the 95% confidence interval.

Joint morphology changes from an initially asymmetric shape to an increasingly symmetrical shape from 3−5 dpf (Fig. 2) and in the absence of muscle contraction joint shape remains asymmetric with the medial side of the MC joint protruding over the PQ (Figs. 2 and 3). Minimum strains are located on the medial side during mouth opening. We, therefore, tested whether cells change their orientation over time to reflect the pattern of strain on the joint and interzone (Figs. 4 and 5). As the joint surface of the MC is more plastic than the surface of the PQ (Fig. 3), we only considered cells from this element. During normal joint morphogenesis, cells on the joint surface of the MC did not show a significant change in cell orientation, apart from those located at the medial side (Fig. 6A, Supp. Table 4). However, for zebrafish that were anaesthetised for 3–5 dpf (Fig. 6B) or *myod* mutants (Fig. 6C), a significant change in orientation in cells on the medial side of the joint was observed at 5 dpf compared to the 5 dpf control medial cell orientations (Supp. Table 4).

## 4 Discussion

Here we present data describing the morphology of the developing zebrafish jaw joint over time, and the effect of removing muscle loads and subsequent biomechanical strain on joint morphology. Normal, wild type joint morphology changes from an initial flattened shape to an interlocking morphology between 3 and 5 dpf. Building on previous work from mouse and chick models showing that joint formation and integrity require muscle activity during development (Drachman and Sokoloff, 1966; Kahn et al., 2009; Nowlan et al., 2010; Roddy et al., 2011b), we show muscle activity is critical for refining the morphology of the zebrafish jaw joint. Critically, our *myod* mutant and anaesthetisation experiments demonstrate that muscle activity is required for joint integrity, rather than the presence of muscle per se. By performing temporal experiments in which we anaesthetise fish for differing time periods we show early muscle activity (from 3 to 4 dpf) is dispensable for normal joint morphogenesis, but movement during later time periods is required for normal joint shape. Therefore the joint retains a degree of plasticity such that loss of early muscle activity does not lead to significantly altered morphology, similar to the situation in chick (Nowlan et al., 2014). In part this may be because muscle activity does not peak until day 4, therefore fewer mechanical cycles will have been experienced during this period. Alternatively, recovery of muscle activity could be sufficient to recover altered joint shape.

The MC surface of the jaw joint is more affected by muscle paralysis than the PQ, suggesting the PQ is less mechanosensitive than the MC. Differential mechanosensitivity of bones has previously been described in limbs (Nowlan et al., 2008) and the mandible (Enomoto et al., 2014).

Our models represent the first FE models for the zebrafish craniofacial skeletal system. Zebrafish are increasingly used as a model for biomechanics, but so far these studies have focused mainly on the ontogeny of swimming behaviour (Fiaz et al., 2012; Fontaine et al., 2008; Green et al., 2011; Li et al., 2012), though studies exist that describe the onset of different jaw movements for respiration and feeding (Hernandez et al., 2002., 2005) and the description of early cell behaviour in forming the jaw elements is well described (Eames et al., 2013; Talbot et al., 2010). Our models show that the strains occurring in the interzone, which drive the changes to zebrafish joint shape are similar in magnitude (up to +3500 µ strain) to those seen in comparable models such as embryonic chick limb (Nowlan et al., 2008), and those used in vitro to elicit cell behaviour changes that drive joint cavitation (Dowthwaite et al., 2003., 1999). Higher strains were generated at constraints and muscle attachment points, but are unlikely to have a significant impact on the model, as the location rather than magnitude of strain is primarily studied.

We show that cell orientation within the joint is significantly affected in fish subjected to a period of immobility. This demonstrates that the cells are altering their behaviour in a strain dependant fashion. Cells on the medial side of the joint change their orientation over time partially accounting for the natural change in joint shape; whereas, in joints where biomechanical strains are reduced, medial cells fail to adopt the correct orientation, leading to an overlapped, non-functional joint. The role of strain in mediating cell orientation in vitro is well characterised for many cell types including chondrocytes (Ghezzi et al., 2014) and ex vivo (Clark et al., 2003).

The FE models show that strains during mouth closure are higher than those during opening. During jaw closure minimum (compressive) principal strains are higher than maximum strains and predominantly seen laterally, loss of strain during immobilisation could lead to a change in cellular processes on the lateral side, with a secondary effect on the medial side. Alternatively, changes to strain location may arise in the morphologically altered immobilised jaws.

During mouth opening peak minimum strains are located at the medial side of the joint interzone. The loss of compression at the medial side of the joint and interzone, could explain the change in chondrocyte behaviour that leads to a switch in cell orientation at the medial side in anaesthetised and *myod* mutant zebrafish. The change in orientation, while significantly contributing to the change in joint shape does not completely explain it, suggesting that there is an additional component, which could be a change to rates of differentiation, proliferation, or migration of chondroprogenitors of the joint. Indeed the failure of chondrocytes to mature fully and intercalate has been previously reported (Shwartz et al., 2012).

We believe that adding 3-dimensional modelling of the joints using FEA to this emerging field will further increase the utility of the zebrafish model for biomechanical studies. Better description of the ontology of the skeletal system that relates zebrafish to higher vertebrate models (Dahdul et al., 2012) will also facilitate future comparisons between model organisms for skeletal biology. Excitingly, use of the zebrafish model with its genetic amenability, opens up the possibility of directly manipulating mechanosensitive genes using transgenic tools to switch genes on or off in the skeletal system, unpicking the relationship between genes and biomechanical influences in shaping a joint.

## Conflict of interest statement

All authors of this manuscript state that they have no conflicts of interest to report. The funders of the study (all of whom are listed in the acknowledgements section of the manuscript) were in no way involved in the experimental design or in the writing of the manuscript.

## Figures and Tables

**Table 1:** The maximum jaw displacement at 5 dpf in 1 min. The average jaw displacement is 37.2 µm.

5 dpf	Max. jaw displacement (µm)
Fish 1	30.5
Fish 2	40.2
Fish 3	37.7
Fish 4	25.6
Fish 5	57.0
Fish 6	31.9

**Table 2:** Muscle forces at (40 nN/µm^2^). 50% of maximum muscle force was applied to the models.

	Number of muscle fibres	Muscle fibre area (µm^2^)	Muscle group area (µm^2^)	Force (*N*)
3 dpf Intermandibularis anterior	5	22.1	110.5	4.42e−6
4 dpf Intermandibularis anterior	5	23.8	119	4.76e−6
5 dpf Intermandibularis anterior	5	23.8	119	4.76e−6
3 dpf Protractor hyoideus	6	22.1	132.6	5.30e−6
4 dpf Protractor hyoideus	6	23.8	142.8	5.71e−6
5 dpf Protractor hyoideus	6	23.8	142.8	5.71e−6
3 dpf Adductor mandibulae	9	22.1	198.9	7.96e−6
4 dpf Adductor mandibulae	9	23.8	214.2	8.57e−6
5 dpf Adductor mandibulae	9	23.8	214.2	8.57e−6
