# Supplementary material for: Finite element modelling predicts changes in joint shape and cell behaviour due to loss of muscle strain in jaw development
Source: J Biomech. 2015 Sep 18;48(12):3112–22. doi: 10.1016/j.jbiomech.2015.07.017 (PMC4601018; doi:10.1016/j.jbiomech.2015.07.017)
Supplement: Supplementary file 1 — Supplementary material [file mmc1.doc]

**Supp. Fig. 1.** Hypermesh models showing muscle attachments and constraint positions. 50% of maximum muscle loads (measured in Newtons (N)) are applied at the origin and insertion of the adductor mandibulae (AM) in the 3dpf (A), 4dpf (C), and 5dpf (E) zebrafish jaw model of mouth closure. Muscle loads at the origin and insertion of the intermandibularis (IM) and protractor hyoideus (PH) in the 3dpf (B), 4dpf (D), and 5dpf (F) zebrafish jaw model of mouth opening. White lines indicate the muscle line of action and white dots indicate the area of applied constraints in either the x,y, or z orientation (as indicated by the orientation in supp. fig 1A).

**Supp. Fig. 2.** The method for identifying cells of the jaw joint for imageJ cell orientation analysis. (A): images of the jaw joint (immunostained for type 2 collagen – using antibody II-II6B3) were loaded into imageJ. Multiple Z projections containing 3 images per stack were produced for each jaw joint image to pick out cells. (B): A threshold was set for each z projection to pick out individual cells (seen in red) within the joint. Cells not within the Meckel’s cartilage joint region (i.e. above the neck of the joint or in the pq) were discounted. Z-stacks were manually examined slice by slice to ensure all relevant cells were included in the analysis. Cells were selected for analysis based on their position within the joint region (which we defined as ending in the ‘neck’ where cells first intercalate). The angle of the joint axis from the horizontal axis was measured in ImageJ (as illustrated with the black angle). (C): The angle of cell orientation was measured in ImageJ in relation to the horizontal axis. Data exported into excel were corrected so that the cell angle was in relation to the joint axis. Medial cells are located within two cells of the inside edge of the MC whilst ‘lateral’ cells are located within two cells of the outer edge. All remaining cells are classified as “central”. 0° was set at the medial (M) and 180° was set at the lateral (L) side of the joint.

**Supp. Fig. 3.** Identification of Meckel’s cartilage and palatoquadrate rudiments.(A): Lateral view of a *Tg(Col2a1aBAC:mcherry)* labelled joint anaesthetised from 5-5+dpf. MC= Meckel’s cartilage, PQ= palatoquadrate, IZ= interzone. A black box on the 3-5+dpf timeline indicates anaesthetisation by MS222 and an empty box indicates no MS222 treatment.

**Supp. Fig. 4.** 3dpf *myod* mutant zebrafish jaw . 3dpf wild type *myod* sibling (A) and 3dpf *myod* mutant (B) zebrafish jaws (for which genotype was confirmed by PCR and sequencing) stained with antibody II-II6B3 for type 2 collagen.

**Supp. Fig. 5.** Finite element models of the 5dpf zebrafish jaw show points of strain exceeding 0.035% are only seen at points where the model is constrained and at muscle attachment points. (A): Finite element model of maximum principal strain on a 5dpf zebrafish jaw visualised to reveal strain above +3500µtrain; all strains are below +3500µstrain (consistent with other models for embryonic cartilage) at the joints and throughout the model except at points of muscle attachment and constraints. (B): Finite element model of minimum principal strain on a 5dpf zebrafish jaw. All strain is below -5000µstrain throughout the model with the exception of model constraints, points of muscle and 3 small regions on the lateral surface of the palatoquadrate.

Supplementary Table 1: FE-model details

|  | No. of elements | No. of nodes | No. of constrained nodes |
| --- | --- | --- | --- |
| 3dpf | 1,269,110 | 259,497 | 35 |
| 4dpf | 1,423,362 | 293,844 | 35 |
| 5dpf | 1,566,355 | 322,186 | 35 |

Supplementary Table 2: Number of cells at each joint region tested for cell orientation at the joint (see Fig. 6).

| Joint area | Number of cells |
| --- | --- |
| 3dpf Medial | 22 |
| 3dpf Central | 12 |
| 3dpf Lateral | 20 |
| 4 dpf Medial | 21 |
| 4 dpf Central | 16 |
| 4 dpf Lateral | 19 |
| 5 dpf Medial | 24 |
| 5 dpf Central | 12 |
| 5 dpf Lateral | 19 |
| 3 dpf Anaes Medial | 28 |
| 3 dpf Anaes Central | 25 |
| 3 dpf Anaes Lateral | 30 |
| 4 dpf Anaes Medial | 27 |
| 4 dpf Anaes Central | 18 |
| 4 dpf Anaes Lateral | 12 |
| 5 dpf Anaes Medial | 30 |
| 5 dpf Anaes Central | 19 |
| 5 dpf Anaes Lateral | 19 |
| 5 dpf *myod* mutant Medial | 15 |
| 5 dpf *myod* mutant Central | 11 |
| 5 dpf *myod* mutant Lateral | 3 |

Supplementary Table 3: p values for joint gap sizes (see Fig. 2F) after Kruskal-Wallis statistical tests. (p≤0.05). (M= medial joint, L= lateral joint, *myod*= *myod* mutant, Anaes= anaesthetised).

|  | 3dpf  M | 3dpf  L | 4dpf  M | 4dpf  L | 5dpf  M | 5dpf  L | 5dpf *myod* M | 5dpf *myod* L | 5dpf Anaes.  M | 5dpf  Anaes.  L |
| --- | --- | --- | --- | --- | --- | --- | --- | --- | --- | --- |
| 3dpf  M | n/a | 0.000 | 0.004 | n/a | 0.055 | n/a | 0.286 | n/a | 0.455 | n/a |
| 3dpf  L | n/a | n/a | n/a | 0.003 | n/a | 0.006 | n/a | 0.475 | n/a | 0.538 |
| 4dpf  M | n/a | n/a | n/a | 0.895 | 0.372 | n/a | 0.002 | n/a | 0.001 | n/a |
| 4dpf  L | n/a | n/a | n/a | n/a | n/a | 0.924 | n/a | 0.005 | n/a | 0.047 |
| 5dpf  M | n/a | n/a | n/a | n/a | n/a | 0.290 | 0.015 | n/a | 0.013 | n/a |
| 5dpf  L | n/a | n/a | n/a | n/a | n/a | n/a | n/a | 0.008 | n/a | 0.067 |
| 5dpf  *myod* M | n/a | n/a | n/a | n/a | n/a | n/a | n/a | 0.000 | 0.654 | n/a |
| 5dpf  *myod* L | n/a | n/a | n/a | n/a | n/a | n/a | n/a | n/a | n/a | 0.258 |
| 5dpf Anaes. M | n/a | n/a | n/a | n/a | n/a | n/a | n/a | n/a | n/a | 0.000 |
| 5dpf Anaes. L | n/a | n/a | n/a | n/a | n/a | n/a | n/a | n/a | n/a | n/a |

Supplementary Table 4: Kruskal-Wallis statistical test p values for cell orientations at the joint (see Fig. 5). (p≤0.05). Red numbers indicate p values of comparisons relevant to the results. (M= medial, C= Central, L=lateral, Con= Control, An= Anaesthetised, *myod* = *myod* mutant)

|  | 3dpf Con  M | 3dpf Con  C | 3dpf Con L | 3dpf An M | 3dpf An  C | 3df An L | 4dpf Con M | 4dpf Con C | 4dpf Con L | 4dpf An M | 4dpf An  C | 4dpf A  L | 5dpf Con  M | 5dpf Con C | 5dpf Con L | 5dpf An M | 5dpf An  C | 5dpf An L | 5dpf *myod* M | 5dpf *myod* C | 5dpf *myod* L |
| --- | --- | --- | --- | --- | --- | --- | --- | --- | --- | --- | --- | --- | --- | --- | --- | --- | --- | --- | --- | --- | --- |
| 3dpf Con M | n/a | 0.015 | 0.030 | 0.067 | 0.000 | 0.010 | **0.695** | 0.220 | 0.077 | 0.935 | 0.358 | 0.672 | n/a | 0.022 | 0.133 | 0.029 | 0.147 | 0.082 | 0.290 | 0.310 | 0.977 |
| 3dpf Con  C | n/a | n/a | n/a | n/a | 0.400 | n/a | n/a | n/a | n/a | n/a | n/a | n/a | n/a | n/a | n/a | n/a | n/a | n/a | n/a | n/a | n/a |
| 3dpf Con L | n/a | 0.583 | n/a | n/a | 0.098 | 0.851 | n/a | n/a | n/a | n/a | n/a | n/a | n/a | 0.674 | n/a | n/a | n/a | n/a | n/a | n/a | n/a |
| 3dpf An M | n/a | 0.313 | 0.614 | n/a | 0.019 | 0.442 | n/a | n/a | 0.917 | n/a | n/a | n/a | n/a | 0.383 | n/a | 0.735 | n/a | 0.939 | n/a | n/a | n/a |
| 3dpf An  C | n/a | n/a | n/a | n/a | n/a | n/a | n/a | n/a | n/a | n/a | n/a | n/a | n/a | n/a | n/a | n/a | n/a | n/a | n/a | n/a | n/a |
| 3dpf An  L | n/a | 0.669 | n/a | n/a | 0.103 | n/a | n/a | n/a | n/a | n/a | n/a | n/a | n/a | 0.772 | n/a | n/a | n/a | n/a | n/a | n/a | n/a |
| 4dpf Con M | n/a | 0.038 | 0.078 | 0.163 | 0.000 | 0.033 | n/a | 0.393 | 0.171 | n/a | 0.591 | 0.928 | n/a | 0.052 | 0.267 | 0.084 | 0.291 | 0.179 | 0.487 | 0.492 | n/a |
| 4dpf ConC | n/a | **0.221** | 0.426 | 0.703 | 0.017 | 0.299 | n/a | n/a | 0.657 | n/a | n/a | n/a | n/a | **0.271** | 0.841 | 0.501 | 0.881 | 0.675 | n/a | n/a | n/a |
| 4dpf Con L | n/a | 0.390 | **0.716** | n/a | 0.044 | 0.560 | n/a | n/a | n/a | n/a | n/a | n/a | n/a | 0.464 | n/a | 0.843 | n/a | n/a | n/a | n/a | n/a |
| 4dpf An  M | n/a | 0.015 | 0.028 | 0.064 | 0.000 | 0.008 | 0.741 | 0.229 | 0.077 | n/a | 0.377 | 0.711 | n/a | 0.021 | 0.135 | 0.027 | 0.151 | 0.081 | 0.303 | 0.325 | n/a |
|  | 3dpf Con  M | 3dpf Con  C | 3dpf Con L | 3dpf An M | 3dpf An  C | 3df An L | 4dpf Con M | 4dpf Con C | 4dpf Con L | 4dpf An M | 4dpf An  C | 4dpf A  L | 5dpf Con  M | 5dpf Con C | 5dpf Con L | 5dpf An M | 5dpf An  C | 5dpf An L | 5dpf *myod* M | 5dpf *myod* C | 5dpf *myod* L |
| 4dpf An  C | n/a | 0.121 | 0.245 | 0.446 | 0.005 | 0.147 | n/a | 0.748 | 0.427 | n/a | n/a | n/a | n/a | 0.154 | 0.587 | 0.284 | 0.624 | 0.442 | 0.858 | 0.829 | n/a |
| 4dpf An  L | n/a | 0.078 | 0.156 | 0.283 | 0.004 | 0.094 | n/a | 0.511 | 0.276 | n/a | 0.707 | n/a | n/a | 0.100 | 0.387 | 0.179 | 0.413 | 0.286 | 0.601 | 0.593 | n/a |
| 5dpf Con M | **0.020** | 0.000 | 0.000 | 0.000 | 0.000 | 0.000 | **0.007** | 0.001 | 0.000 | 0.011 | 0.002 | 0.018 | n/a | 0.000 | 0.000 | **0.000** | 0.000 | 0.000 | **0.002** | 0.004 | 0.251 |
| 5dpf Con C | n/a | **0.908** | n/a | n/a | 0.329 | n/a | n/a | n/a | n/a | n/a | n/a | n/a | n/a | n/a | n/a | n/a | n/a | n/a | n/a | n/a | n/a |
| 5dpf Con L | n/a | 0.278 | **0.534** | 0.863 | 0.022 | 0.387 | n/a | n/a | **0.799** | n/a | n/a | n/a | n/a | 0.339 | n/a | 0.632 | n/a | **0.819** | n/a | n/a | n/a |
| 5dpf An  M | n/a | 0.448 | 0.839 | n/a | 0.040 | 0.662 | n/a | n/a | n/a | n/a | n/a | n/a | n/a | 0.534 | n/a | n/a | n/a | n/a | n/a | n/a | n/a |
| 5dpf An  C | n/a | 0.258 | 0.500 | 0.817 | 0.019 | 0.356 | n/a | n/a | 0.759 | n/a | n/a | n/a | n/a | **0.316** | 0.958 | 0.591 | n/a | 0.778 | n/a | n/a | n/a |
| 5dpf An  L | n/a | 0.378 | 0.697 | n/a | 0.041 | 0.541 | n/a | n/a | 0.980 | n/a | n/a | n/a | n/a | 0.450 | n/a | 0.822 | n/a | n/a | n/a | n/a | n/a |
| 5dpf *myod* M | n/a | 0.183 | 0.356 | 0.600 | 0.013 | 0.242 | n/a | 0.893 | 0.565 | n/a | n/a | n/a | n/a | 0.226 | 0.736 | 0.417 | 0.774 | 0.581 | n/a | 0.959 | n/a |
| 5dpf *myod* C | n/a | 0.235 | 0.432 | 0.679 | 0.029 | 0.322 | n/a | 0.944 | 0.638 | n/a | n/a | n/a | n/a | **0.283** | 0.800 | 0.503 | 0.836 | 0.653 | n/a | n/a | n/a |
| 5dpf *myod* L | n/a | 0.186 | 0.292 | 0.406 | 0.060 | 0.243 | 0.869 | 0.541 | 0.389 | 0.993 | 0.660 | 0.835 | n/a | 0.212 | **0.466** | 0.327 | 0.483 | 0.396 | 0.594 | 0.583 | n/a |
